# ZrSnO_4_: A Solution-Processed Robust Electron Transport Layer of Efficient Planar-Heterojunction Perovskite Solar Cells

**DOI:** 10.3390/nano11113090

**Published:** 2021-11-16

**Authors:** Jun Choi, Young Ki Park, Hee Dong Lee, Seok Il Hong, Woosung Lee, Jae Woong Jung

**Affiliations:** 1 Material & Component Convergence R&D Department, Korea Institute of Industrial Technology (KITECH), Ansan-si 15588, Gyeonggi-do, Korea; skywork1@kitech.re.kr; 2 Advanced Textile R&D Department, Korea Institute of Industrial Technology (KITECH), Ansan-si 15588, Gyeonggi-do, Korea; parkyk@kitech.re.kr (Y.K.P.); lhd0121@kitech.re.kr (H.D.L.); redstone@kitech.re.kr (S.I.H.); 3 Integrated Education Institute for Frontier Materials (BK21 Four), Kyung Hee University, 1732 Deogyeong-daero, Giheung-gu, Yongin-si 446-701, Gyeonggi-do, Korea; 4 Department of Advanced Materials Engineering for Information and Electronics, Kyung Hee University, 1732 Deogyeong-daero, Giheung-gu, Yongin-si 446-701, Gyeonggi-do, Korea

**Keywords:** ZrSnO_4_, sol-gel synthesis, perovskite solar cells, electron transport layer

## Abstract

A robust electron transport layer (ETL) is an essential component in planar-heterojunction perovskite solar cells (PSCs). Herein, a sol-gel-driven ZrSnO_4_ thin film is synthesized and its optoelectronic properties are systematically investigated. The optimized processing conditions for sol-gel synthesis produce a ZrSnO_4_ thin film that exhibits high optical transmittance in the UV-Vis-NIR range, a suitable conduction band maximum, and good electrical conductivity, revealing its potential for application in the ETL of planar-heterojunction PSCs. Consequently, the ZrSnO_4_ ETL-based devices deliver promising power conversion efficiency (PCE) up to 19.05% from CH_3_NH_3_PbI_3_-based planar-heterojunction devices. Furthermore, the optimal ZrSnO_4_ ETL also contributes to decent long-term stability of the non-encapsulated device for 360 h in an ambient atmosphere (*T*~25 °C, *RH*~55%,), suggesting great potential of the sol-gel-driven ZrSnO_4_ thin film for a robust solution-processed ETL material in high-performance PSCs.

## 1. Introduction

As the world is heading towards global environmental issues and an energy crisis, the utilization of renewable energy resources has attracted tremendous attention. In specific, solar energy conversion is the most practical sustainable energy resource and has thus become the fastest growing and most affordable source of electricity generation [1]. Recently, the organic–inorganic metal halide perovskites have emerged as a game changer in the field of photovoltaics due to their reduced processing cost and high-power conversion efficiency [2,3,4,5]. Numerous benefits of perovskite solar cells (PSCs), such as their strong absorption coefficient, favorable bandgap, long carrier lifetime, and high charge carrier mobility, make them the most promising photovoltaic technology for the coming generations [6,7,8,9,10,11]. In the past few years, the photovoltaic properties of PSCs have been boosted by advancements in materials engineering and device optimization, and the fruit of this effort has been realized by excellent power conversion efficiency (PCE) to >25% in a single-junction device [12].

In addition to engineering of the photoactive perovskite layer, interface optimization also plays a crucial role in determining device performance of PSCs because surface composition, crystal orientation, lattice phase, and film morphology of the perovskite absorber are highly dependent on the interfacial properties of the device [13,14,15]. Currently, titanium oxide (TiO_2_) is the most common electron transport layer (ETL) material for fabricating high-performance PSCs. Due to its excellent electrical conductivity and large band-gap, effective electron extraction and hole blocking have been demonstrated at the interface of the perovskite absorber layer and cathode in the normal-structured (*n-i-p*) devices [16,17,18,19,20]. In order to achieve high electrical properties, however, the mesoporous structure must be prepared for the TiO_2_ ETL; this requires sintering at >400 °C via a complicated deposition process (e.g., spray pyrolysis), thus it is costly and cannot be applied to a flexible substrate. Recently, *n*-type metal oxides such as tin(IV) oxide (SnO_2_) have been employed in planar-heterojunction architecture as a compact ETL to replace TiO_2_-based ETLs, because of their potential as scalable and flexible devices with low fabrication costs [21]. SnO_2_ is an appealing ETL in PSCs due to its suitable energy level alignment to the perovskite absorber, decent electron mobility, large band-gap, and outstanding stability under light, heat, and moisture. However, SnO_2_ might have defective sites, such as oxygen vacancy on the surface, which may deteriorate the durability of device. Furthermore, the electronic structure of the interfaces in multi-layered optoelectronic devices may govern charge transport, extraction, and recombination kinetics, so novel *n*-type materials with improved electronic properties should be explored to further optimize the interfacial properties and charge transport behavior of PSCs [22].

In recent years, ternary metal oxides (TMOs) have been regarded as a novel oxide semiconductor for use in optoelectronic applications [23]. Due to tunable optical and electronic properties, TMOs possess controlled charge transport properties which thus make them suitable for charge transporting or extracting materials for solar cell devices [24,25,26]. In particular, the Sn-containing TMOs have been studied in the field of photovoltaics due to their optical transparency, decent conductivity, and chemical stability; Kim et al. studied a BaSnO_3_–based photoanode in dye-sensitized solar cells with a promising PCE of 5.2% [27] while Lee et al. reported a Zn_2_SnO_4_—based photoelectrode in the mesoscopic device of the PSCs [28]. Meanwhile, we previously reported on the room-temperature synthesis of ZrSnO_4_ nanoparticles (NPs) and their application in PSCs as an ETL [29]. The tailored electronic properties of ZrSnO_4_ NPs, such as high electrical conductivity, optical transparency, and balanced energy levels to the perovskite absorber, delivered promising photovoltaic properties of PSCs with a PCE up to 16.76%. However, despite the potential of ZrSnO_4_ as an ETL in PSCs, the colloidal NPs of TMOs may have some issues, such as a large number of surface defects, complexity and low yield of the synthetic process, batch-to-batch variation of NPs, and difficulties in electronic structure control of NPs according to size variation [30]. On the contrary, the sol-gel synthesis of metal oxides involves formation of sol from a homogeneously mixed solution, converting them into gel by a polycondensation process and finally a heating process according to the material required. The sol-gel process provides a reliable, scalable, reproducible, and cost-effective way to prepare high-quality metal oxide thin films, and would be a suitable process for preparing practical ZrSnO_4_ ETLs over NPs for high-performance PSCs.

In this work, we propose a sol-gel synthesis of ZrSnO_4_ thin films for preparing a robust ETL in planar-heterojunction PSCs. The optoelectronic properties of the ZrSnO_4_ ETL were optimized with respect to annealing temperature per our design. The sol-gel-driven ZrSnO_4_ ETL exhibited excellent optical transmittance, decent electrical conductivity, and a favorable energy level for extracting electrons from the conduction band of the perovskite absorber layer. The CH_3_NH_3_PbI_3_-based solar cell devices employing the optimized ZrSnO_4_ ETL achieved a PCE up to 19.05% with good long-term stability in ambient conditions under continuous 1-sun illumination. Systematic device analyses concluded that the sol-gel-driven ZrSnO_4_ ETL renders potential for fast electron extraction and effective hole blocking, which promises a robust ETL candidate for high-performance PSCs and other photoelectric devices.

## 2. Materials and Methods

### 2.1. Materials

All reagents and solvents were obtained from commercial sources (Sigma-Aldrich, St. Louis, MO, USA and Sejin CI, Seoul, Republic of Korea) at reagent-grade and used as received. The precursor solution of ZrSnO_4_ was prepared by mixing 1.05 mL of SnCl_2_ (1 M in 2-butanol), 1.05 mL of ZrOCl (1 M in 2-butanol), 0.005 mL of HNO_3_, and 0.085 mL of acetylacetone thoroughly in a vial. Ethanol (0.35 mL), 2-butanol (0.35 mL), and deionized water (0.1 mL) were added to the mixture to dilute the precursor solution. After the solution was further stirred at room temperature for 0.5 h it was filtered by PVDF filters (pore size = 0.45 μm). The perovskite precursor solution was prepared by dissolving CH_3_NH_2_I (224.2 mg) and PbI_2_ (650 mg) in anhydrous dimethylformamide (0.897 mL). Anhydrous dimethyl sulfoxide (0.1 mL) was then added to the solution. The precursor solution was filtered by PTFE-D filters (pore size = 0.2 μm) after vigorous stirring at room temperature for 10 min.

### 2.2. Fabrication of Thin-Film Perovskite Solar Cells

The indium tin oxide (ITO)-coated glass substrates (sheet resistance~20 Ω/sq) were cleaned sequentially with water, acetone, and isopropanol under sonication for 30 min, and then treated with UV-O_3_ for over 30 min. After the ZrSnO_4_ layer was formed by spin-coating the ITO-coated glass (3000 RPM for 30 s), the substrate was annealed in an ambient atmosphere for 1 h at a specified temperature (200 to 400 °C). The substrates were then cooled down to room temperature to deposit the perovskite layer by spin-coating the precursor solution at 3000 rpm for 30 s. After 2 s, 400 µL of Diethyl eher was poured on top of the substrates during spin-coating. After the perovskite layer was annealed at 150 °C for 5 min, the Spiro-OMeTAD layer was deposited by spin-coating the solution in chlorobenzene (90.9 mg mL^−1^) with 23 μL of bis(trifluoromethane)sulfonamide lithium salt in acetonitrile (540 mg mL^−1^) and 4-tert-butylpyridine (39 μL). Finally, 80 nm of Au was evaporated under high vacuum (<5 × 10^−6^ Torr). The device area was defined by the shadow mask as 10 mm^2^.

### 2.3. Characterization

Optical properties including absorptivity and transparency were measured using a spectrometer (Cary 100, Agilent, CA, USA). The surface morphology of the films was recorded using an atomic force microscope (CoreAFM, Nanosurf, Liestal, Switzerland) in tapping mode and scanning electron microscopy (S-4800, Hitach) at the Core Facility Center for Analysis of Optoelectronic Materials and Devices of the Korea Basic Science Institute (KBSI). The *J−V* curves were recorded using a SourceMeter (2400-SCS, Keithley, OR, USA) under the illumination of an AM 1.5 G-simulated light source (100 mW cm^−2^). The light intensity was calibrated using a NREL-certified photodiode. The incident photon-to-current density efficiency was measured using a lock-in amplifier system which records the short-circuit current density under chopped monochromatic light.

## 3. Results

**Figure 1:**
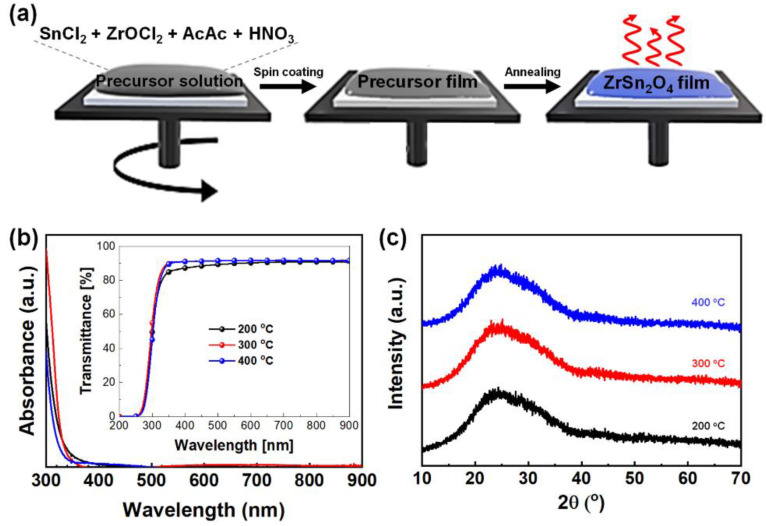
Synthetic scheme (**a**), absorption spectra (**b**), and X-ray diffractograms (**c**) of ZrSnO_4_ films prepared by sol-gel method.

The sol-gel-driven ZrSnO_4_ thin film was prepared from the precursor solution of SnCl_2_ and ZrOCl in the mixed solvent system (deionized water, ethanol, and 2-butanol), as described in Materials and Methods. Acetylacetone and nitric acid were added to the solution to accelerate the hydrolysis process of the metal precursors [31]. As illustrated in Figure 1a, the prepared precursor solution of ZrSnO_4_ film was deposited over the substrate by spin-coating, followed by thermal annealing at a varied temperature (200 to 400 °C) to optimize the film quality of the ZrSnO_4_ layer on charge transport in devices. The resulting ZrSnO_4_ films were clear and transparent after thermal annealing for 1 h, as shown in Figure S1. The absorption spectroscopy measurement displayed in Figure 1b shows that the films exhibit limited absorption in the UV region (less than 400 nm). The optical band-gap of the ZrSnO_4_ film was 2.93 eV, according to the Tauc plot (Figure S2). Because the photons arrive at the perovskite absorbance layer through the ETLs in the normal-structured device, sufficient optical transparency is a prerequisite for achieving excellent photon absorption and power output. As shown in the inset of Figure 1b, the transmittance spectra of the ZrSnO_4_ films exhibit high transparency in the UV-visible wavelength range, which guarantees enough photon harvesting of perovskite absorber films regardless of annealing temperature.

The crystalline properties of ZrSnO_4_ films were characterized in detail using X-ray diffraction (XRD). (Figure 1c) In previous studies, it is reported that ZrSnO_4_ can form a metastable orthorhombic (*Pbcn*) crystalline structure in a temperature range of 600−800 °C. In our samples, we did not observe any crystalline peak of ZrSnO_4_ at the annealing temperature of 200−400 °C [32]. (JCPDS 00-048-0889 shown in Figure S3) Therefore, we confirmed that the ZrSnO_4_ films prepared using the sol-gel method were in an amorphous state up to the 400 °C annealing temperature.

**Figure 2:**
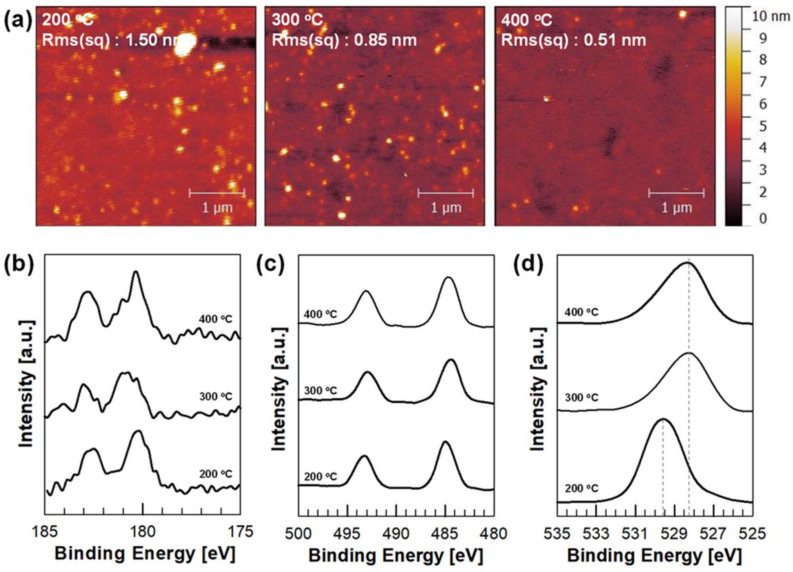
AFM topographic images (**a**) and XPS binding energy spectra for Zr 3d (**b**), Sn 3d (**c**), and O 1s (**d**) of sol-gel-driven ZrSnO_4_ films.

To investigate the surface property of the films upon different annealing temperatures, surface topography of the films was studied using atomic force microscopy (AFM). As displayed in Figure 2a, the sol-gel process allowed a uniform and compact surface morphology of the ZrSnO_4_ thin films. The root-mean-square (RMS) roughness of ZrSnO_4_ films with an annealing temperature of 200, 300, and 400 °C was 1.50, 0.85, and 0.51 nm, respectively, which reveals a very smooth film surface of ZrSnO_4_ thin films without discernible grain boundaries or defects. The ultra-smooth surface of the films was attributed to the amorphous nature of the sol-gel-driven ZrSnO_4_ films, regardless of the annealing temperature. A smooth surface of bottom layers will benefit the complete coverage of the perovskite absorbing layers in the device. The electronic configuration of the sol-gel-driven ZrSnO_4_ film was studied using X-ray photoelectron spectroscopy (XPS). Figure 2b,c present the XPS results of the Zr 3d and Sn 3d, respectively, which confirm the presence of Zr and Sn elements in the ZrSnO_4_ films prepared by sol-gel synthesis. The core level spectra for Zr 3d show two peaks centered at 181 and 183 eV which were originated from Zr 3d_5/2_ and Zr 3d_3/2_ doublet, respectively, while the core levels for Sn 3p_3/2_ and Sn 3p_5/2_ peaks were observed at 493 and 485 eV, respectively. There was no significant shift of the binding energies for Zr 3d and Sn 3d in three samples, which reveals that the electronic structure for Zr and Sn ions does not change as the annealing temperature increases. The broad peak centered at ~530 eV is designated to O 1s. (Figure 2d) In general, the coordinated oxygen atom bonded metal cations exhibited rather lower binding energy as compared to the higher binding energy species owing to oxygen deficiencies [33]. As per earlier reports, the peak shift for O 1s in the ZrSnO_4_ films annealed at higher temperature indicates a more ordered electronic structure in the lattice. In addition, there were less oxygen vacancies or deficiencies, which could contribute to the improved electronic properties of ZrSnO_4_ film.

**Figure 3:**
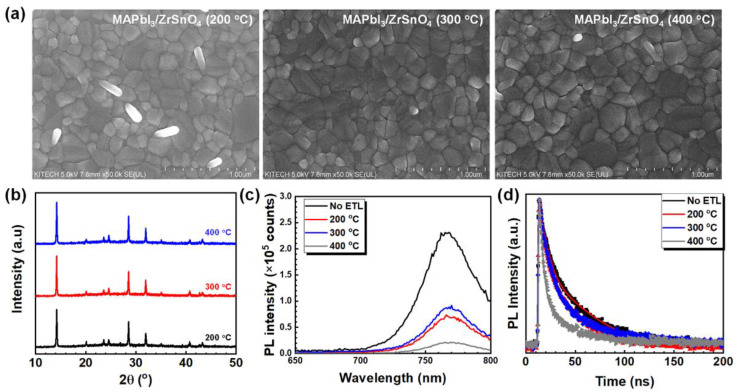
FE-SEM images of the top view (**a**), XRD diffractograms (**b**), PL spectra (**c**), and TRPL curves (**d**) of perovskite absorber layer deposited on the sol-gel-driven ZrSnO_4_ films annealed at different temperatures.

The bottom interface can influence the growth/crystallization of the perovskite layer, which thus influences the optoelectronic properties of the perovskite absorber and the device performance of PSCs. As shown in Figure 3a, the surface morphology of perovskite (CH_3_NH_3_PbI_3_) films deposited on different ZrSnO_4_ thin films looked similar. The SEM images display fully covered perovskite absorber layers with textured morphology, such as compact grains and smooth surface, regardless of the underlying ZrSnO_4_ layer. The X-ray diffractograms for three samples were also comparable with three representative peaks ((110), (220), and (310) at 2*θ* = 14.2, 28.4, and 32.1°, respectively), thus indicating that the annealing temperature of ZrSnO_4_ thin films did not significantly affect film morphology and crystallinity of the perovskite absorber layers. (Figure 3b) However, a few rod-like bright spots existed at the perovskite layer on the ZrSnO_4_ ETL (200 °C) which might be PbI_2_ or defective sites.

Besides the role of the ETL as a robust substrate for growing a high-quality perovskite absorber layer, ETLs play a key role in the device to extract electrons from the perovskite layer and block the recombination between electrons and holes at the interface of ITO and holes in the perovskite layer. The electron extraction and transport properties were studied using steady-state photoluminescence (PL) spectra of perovskite absorber layers grown on different ZrSnO_4_ ETLs, as presented in Figure 3c. The pristine perovskite layer on glass substrate shows an intense PL emission centered at 764 nm, and the PL intensity is markedly reduced as the ZrSnO_4_ films were employed under the perovskite layer, which reveals successful electron extraction from perovskite layer. It is noted that the electron conductivity of ZrSnO_4_ film is 4 × 10^−4^ S/m, which is comparable to crystalline TiO_2_ film (σ~10^−3^–10^−4^ S/m), so the photo-generated electrons would be effectively transferred to the ZrSnO_4_ ETL. (Figure S4) Among the samples, the perovskite deposited onto the ZrSnO_4_ ETL prepared at 400 °C exhibits the least PL intensity when compared to the other films. This is contributed to efficient electron extraction from the perovskite layer to the ETL. The corresponding PL lifetime is assessed using the time-correlated single-photon counting (TCSPC) technique. Figure 3d shows the time-correlated PL decay of the perovskite absorber layers on ZrSnO_4_ ETLs. The average PL lifetime (*τ*_ave_) was extracted by fitting the bi-exponential decay function in which the two relaxation decay pathways are associated with non-radiative decay, such as electron extraction by the ETLs, and radiative decay in the perovskite layer corresponding to fast (*τ*_1_) and slow time constant (*τ*_2_), respectively. As listed in Table S1, the pristine CH_3_NH_3_PbI_3_ layer exhibits *τ*_ave_ of 41.84 ns, while it was substantially decreased as the ZrSnO_4_ ETLs were introduced as an ETL. The perovskite layer showed the *τ*_ave_ of PL in the presence of ZrSnO_4_ ETL: 25.76 ns for 200 °C; 26.38 ns for 300 °C; 8.71 ns for 400 °C. Since the quick PL quenching primarily corresponds to the quick charge extraction from the perovskite absorber, it is concluded that the ZrSnO_4_ ETL prepared at 400 °C would be the most effective ETL for achieving high-performance PSCs.

**Figure 4:**
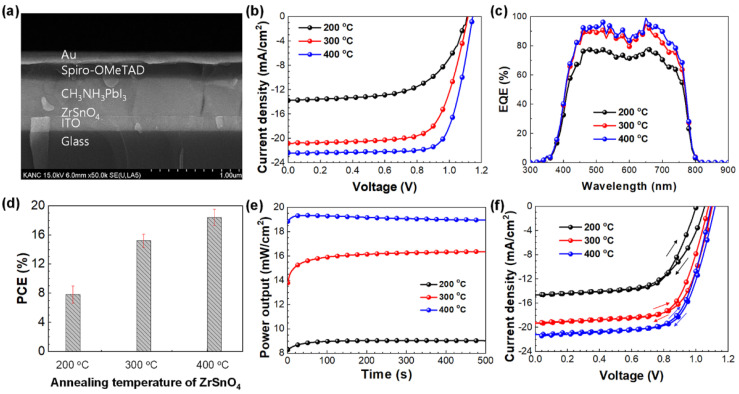
FE-SEM image for the cross-section of device (**a**), *J–V* curves (**b**), EQE spectra (**c**), statistical analysis of PCEs of the devices (**d**), stabilized power output measurements (**e**), and hysteresis analysis of the PSCs employing the sol-gel-driven ZrSnO_4_ ETLs with different temperature (**f**). The statics of PCE data was collected from more than 12 devices in each condition.

Based on the above investigation, the photovoltaic properties of the ZrSnO_4_ ETL-based PSCs were examined. The planar-heterojunction devices were fabricated with an *n*-*i*-*p* structure (ITO/ZrSnO_4_ ETL/CH_3_NH_3_PbI_3_/Spiro-OMeTAD/Au). The cross-sectional SEM image shown in Figure 4a presents well-defined interfaces of the representative device. The photocurrent-voltage (*J−V*) curves of the optimal device in each condition under the simulated illumination of 1-sun condition (100 mW cm^–2^, AM 1.5 G) are depicted in Figure 4b. The corresponding photovoltaic parameters of the devices are summarized in Table 1. As expected, the PCEs of the devices were dependent on the condition of the ZrSnO_4_ ETLs. For the ZrSnO_4_ ETL annealed at 200 °C, the device produced a PCE of 9.19% with a *V*_OC_ of 1.10 V, a *J*_SC_ of 20.82 mA cm^–2^, and a fill factor (FF) of 60.61%. As the annealing temperature increased, the PCEs were enhanced to 16.15% and 19.05% as the ZrSnO_4_ ETL was annealed at 300 and 400 °C, respectively. We also investigated the 500 °C annealing temperature of a ZrSnO_4_ ETL, but it delivered very poor photovoltaic property (PCE less than 1%), as displayed in Figure S5. The *J−V* curve of the 500 °C-annealed ZrSnO_4_ ETL was an S-curve shape, which reveals charge accumulation and recombination due to very poor charge transport and extraction capability of the interfaces of device [34]. Thus, we conclude that high temperature annealing over 400 °C may induce deterioration of the ZrSnO_4_ ETL which degrades device performance of PSCs; thus, 400 °C is the optimal annealing condition for preparing high-quality ZrSnO_4_ ETL for PSCs.

Although *V*_OC_ of the device did not improve significantly, the notable increase in *J*_SC_ and FF of the devices with the ZrSnO_4_ ETLs prepared at 400 °C annealing condition is likely to be related to an improved interface at ITO/ETL/perovskite layer. External quantum efficiency (EQE) spectra of the devices are shown in Figure 4c, with the integrated current densities being 13.52, 20.67, and 21.85 mA cm^–2^ for the devices of ZrSnO_4_ ETLs at 200, 300, and 400 °C annealing temperature, respectively; these values are consistent with the *J*_SC_ values obtained from *J–V* measurements under 1-sun illumination. Figure 4d displays the stabilized maximum power point (MPP) measurement for the devices under illumination for 500 s. For the device with the optimal ZrSnO_4_ ETL (400 °C), the stabilized PCE is estimated to be 18.93%, which is in good agreement with the PCEs obtained from *J–V* measurements. The MPP for the other devices were also well matched with their PCE values. The reproducibility of the ZrSnO_4_ ETLs can also be confirmed by the statistics shown in Figure 4e, indicating that the sol-gel synthesis strategy of ZrSnO_4_ can provide homogeneous but excellent electrical properties for the ETL films. It is noteworthy that the light soaking of the ZrSnO_4_ ETL does not improve the device performance, as shown in Figure S6. Thus, the PCEs summarized in Figure 4d and Table 1 were obtained from freshly prepared devices without light soaking.

Since the PSCs studied in this work consist of planar-heterojunction interfaces of ETLs, we then compared the hysteresis of the *J–V* measurements under 1-sun illumination upon different scan direction, as shown in Figure 4f. Hysteresis index (*HI*) is defined as the Equation (1) to quantify the degree of hysteresis of *J–V* measurements [35].

(1) $$HI=\frac{J_{RS}\left(0.8V_{OC}\right)-J_{FS}\left(0.8V_{OC}\right)}{J_{RS}\left(0.8V_{OC}\right)}$$

In the case of the optimal ZrSnO_4_ ETL (400 °C), there was negligible hysteresis with a hysteresis index (*HI*) of 2.04%, with PCEs of 17.71% and 16.75% for forward and reverse scans, respectively. In contrast, the hysteresis of the devices with ZrSnO_4_ ETLs prepared at a lower temperature was exacerbated (9.61% and 9.07% for ZrSnO_4_ ETL prepared at 200 °C and 300 °C, respectively), as summarized in Table S2. Although the exact role of ZrSnO_4_ ETLs on the hysteresis behavior of the device is not clear, such improved hysteresis of photovoltaic performance for the optimal ETL (ZrSnO_4_ (400 °C)) is likely to be related to improved interfacial properties between ITO/ETL and ETL/perovskite.

**Figure 5:**
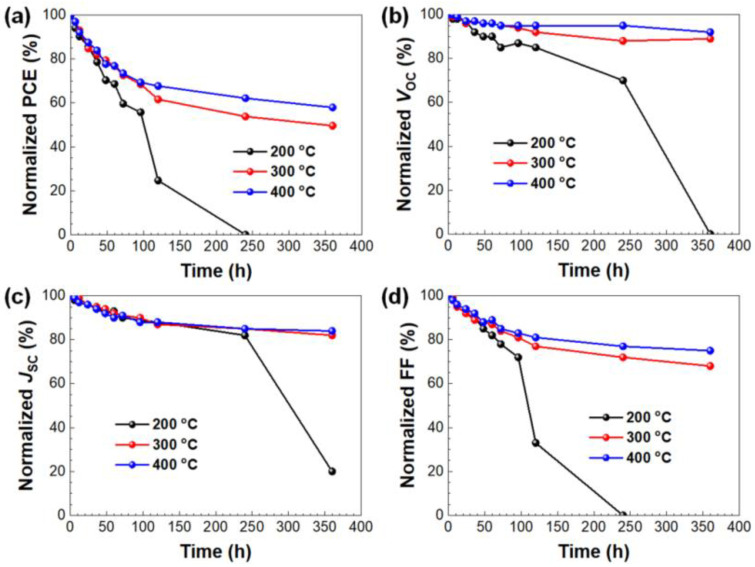
Long-term decay curves of devices in ambient condition: PCE (**a**), *V*_OC_ (**b**), *J*_SC_ (**c**), and FF (**d**).

The environmental stability of the devices was then investigated in an ambient atmosphere (*T*~25 °C; *RH*~55%) under continuous illumination of 1-sun simulated light (100 mW cm^–2^). The long-term stability results are shown in Figure 5. After being exposed to ambient conditions for 360 h, the devices maintained 59% and 50% with ZrSnO_4_ ETLs annealed at 400 °C and 300 °C, respectively, of its original value. On the contrary, the device was degraded to zero PCE after 240 h in the presence of ZrSnO_4_ ETLs (200 °C). One of the reasons leading to the better stability is a much more stable *V*_OC_ and FF in comparison to those of the 200 °C-annealed ETL. On the contrary, *J*_SCS_ for the three devices gradually decreases for an initial 240 h, but the 200 °C-annealed device could not retain photocurrent generation after 240 h ambient storage; this indicates that the major parameters determining device stability are *V*_OC_ and FF. The relatively stable *V*_OC_ and FF values for of ZrSnO_4_ ETLs (300 and 400 °C) benefit from a more stable interface of the ITO/ETL/perovskite layer following optimized electronic properties as compared to the ZrSnO_4_ ETL prepared at 200 °C. As a result, the devices based on ZrSnO_4_ ETLs achieve an excellent long-term stability under continuous illumination conditions, indicating that sol-gel-driven ZrSnO_4_ can be a promising ETL material for highly efficient, reliable, and robust PSCs.

## 4. Conclusions

In summary, we have demonstrated the sol-gel synthesis of ZrSnO_4_ and its application to PSCs as a robust ETL has been addressed. The sol-gel synthesis afforded amorphous ZrSnO_4_ thin films, but its smooth surface and high optical transparency allowed it to be a promising candidate for ETL materials of *n-i-p*-structured PSCs. The electronic structure was optimized by varying the annealing temperature of sol-gel synthesis for the ZrSnO_4_ thin films. The PSCs employing ZrSnO_4_ ETLs achieve high photovoltaic performance of 19.05% efficiency with a *V*_OC_ of 1.12 V, a *J*_SC_ of 22.28 mA cm^–2^, and an FF of 76.34%, indicating the potential of the sol-gel method for synthesizing ZrSnO_4_ ETLs for high-performance PSCs. ZrSnO4 ETLs also delivered outstanding environmental stability for 360 h under continuous 1-sun illumination. Our results will inspire the sol-gel synthesis of metal oxide-based ETL materials for efficient, reliable, and robust perovskite photovoltaics.

## Figures and Tables

**Table 1:** Photovoltaic parameters of the PSCs based on ZrSnO_4_ ETLs with varying annealing temperature.

Annealing Temperature [°C]	*V*_OC_ [V]	*J*_SC_ [mA cm^2^]	*J*_EQE_ ^1^ [mA cm^–2^]	FF [%]	PCE [%]	MPP ^2^ [mW cm^–2^]
200	1.10	13.78	13.52	60.61	9.19	9.03
300	1.10	20.82	20.67	70.52	16.15	16.20
400	1.12	22.28	21.85	76.34	19.05	18.93

^1^ Integrated current density values from EQE graph. ^2^ Maximum power point output under 1-sun illumination for 500 s.

## Supplementary Materials

The following are available online at https://www.mdpi.com/article/10.3390/nano11113090/s1, Figure S1: Photographs of sol-gel-driven ZrSnO_4_ films, Figure S2: Tauc plot, Figure S3: Theoretical XRD for ZrSnO_4_ film, Figure S4: Conductivity measurement, Figure S5: *J−V* curve of device, Figure S6: *J−V* curves of device after light soaking, Table S1: TRPL parameter, Table S2: photovoltaic parameters, Table S3: photovoltaic measurement.
