# Supplementary material for: ZrSnO4: A Solution-Processed Robust Electron Transport Layer of Efficient Planar-Heterojunction Perovskite Solar Cells
Source: Nanomaterials (Basel). 2021 Nov 16;11(11):3090. doi: 10.3390/nano11113090 (PMC8625985; doi:10.3390/nano11113090)
Supplement: Supplementary file 1 [file nanomaterials-11-03090-s001.zip › nanomaterials-1448174-SI.pdf]

nanomaterials

MDPI

Supplementary Materials

$ZrSnO_4$: A Solution-Processed Robust Electron Transport Layer of Efficient Planar-Heterojunction Perovskite Solar Cells

Jun Choi [1], Young Ki Park [2], Hee Dong Lee [2], Seok Il Hong [2], Woosung Lee [2,*] and Jae Woong Jung [3,4,*]

1 Material & Component Convergence R&D Department, Korea Institute of Industrial Technology (KITECH), Ansan 15588, Korea; skywork1@kitech.re.kr
2 Advanced Textile R&D Department, Korea Institute of Industrial Technology(KITECH), Gyeonggi-do 15588, Korea; parkyk@kitech.re.kr (Y.K.P.); lhd0121@kitech.re.kr (H.D.L.); red-stone@kitech.re.kr (S.I.H.)
3 Integrated Education Institute for Frontier Materials (BK21 Four), Kyung Hee University, 1732 Deogyeong-daero, Giheung-gu, Yongin-si, Gyeonggi-do 446-701, Korea
4 Department of Advanced Materials Engineering for Information and Electronics, Kyung Hee University, 1732 Deogyeong-daero, Giheung-gu, Yongin-si, Gyeonggi-do 446-701, Korea
* Correspondence: wslee@kitech.re.kr (W.L.); wodndwjd@khu.ac.kr (J.W.J.); Tel.: +82-31-201-2435

Table S1. Time parameters of PSCs with different $ZrSnO_4$ ETL with different *J*–*V* scan.

Annealing Temperature of $ZrSnO_4$ [°C]	t_1 (ns)	t_2 (ns)	t_{avg} (ns)
–	9.34	62.00	41.84
200	4.88	48.23	25.76
300	4.97	49.40	26.38
400	1.83	20.02	7.71

Table S2. Device parameters of PSCs with different $ZrSnO_4$ ETL with different *J*–*V* scan directions.

Annealing Temperature of $ZrSnO_4$ [°C]	Scan Sirection	V_{OC} [V]	J_{SC} [mA/cm²]	FF	PCE [%]	HI [%]
200	Forward	1.00	14.64	0.66	9.64	9.07
	Backward	1.09	14.65	0.58	9.22	
300	Forward	1.01	19.33	0.73	14.46	9.61
	Backward	1.11	19.28	0.65	14.19	
400	Forward	1.11	21.21	0.75	17.71	2.04
	Backward	1.12	21.45	0.71	16.75	

Table S3. Device parameters of PSCs with light soaking effect for $ZrSnO_4$ ETL (400 °C)-based PSCs.

Light Soaking Time	V_{OC} [V]	J_{SC} [mA/cm²]	FF	PCE [%]
No light soaking	1.09	21.59	0.74	17.52
0.5 min	1.08	21.54	0.73	17.35
1.0 min	1.06	21.27	0.72	16.88

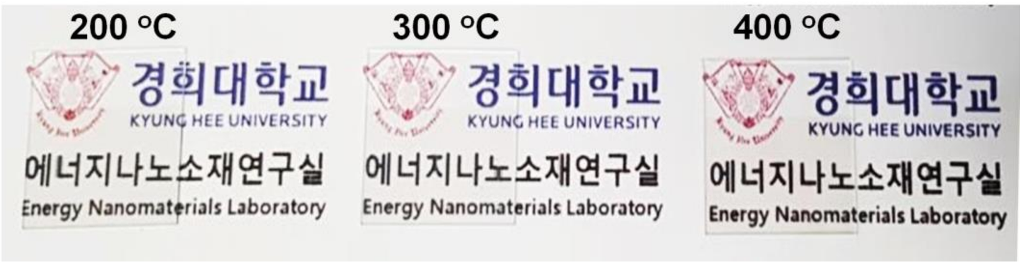

Figure S1. Photographs of sol-gel-driven $ZrSnO_4$ films annealed at 200 °C (left), 300 °C (middle), and 400 °C (right). (Substrate size = 2 × 2 cm^2).

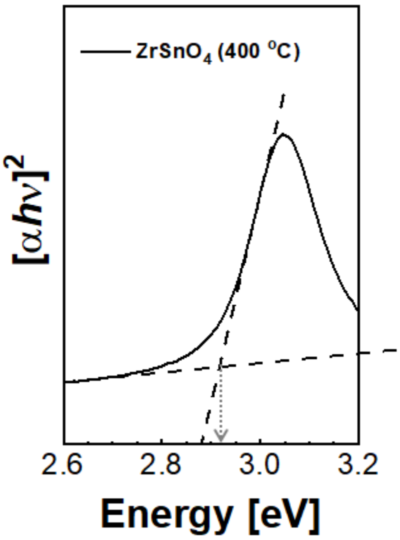

Figure S2. Tauc plot of $ZrSnO_4$ film annealed at 400 °C.

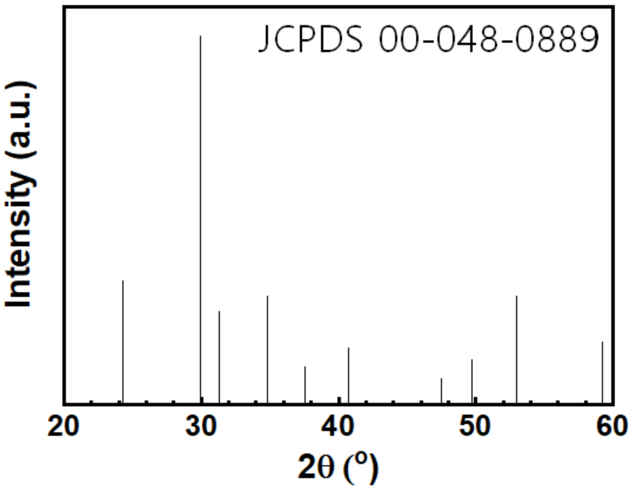

Figure S3. Orthorombic (*Pbcn*) phase of $ZrSnO_4$ (JCPDS 00-048-0889).

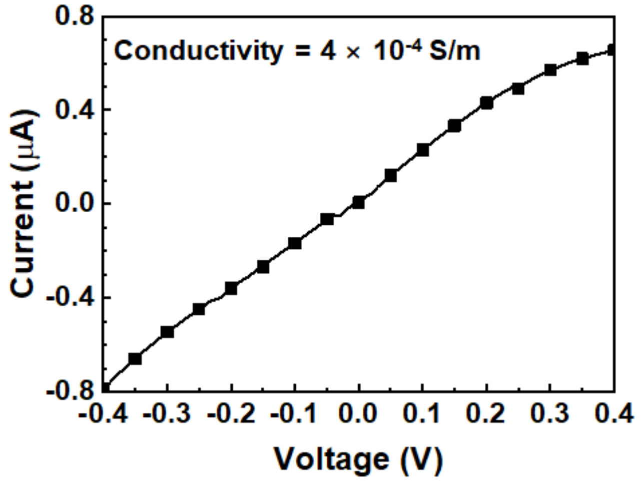

Figure S4. *I*–*V* curve of $ZrSnO_4$ (400 °C) film for conductivity measurement.

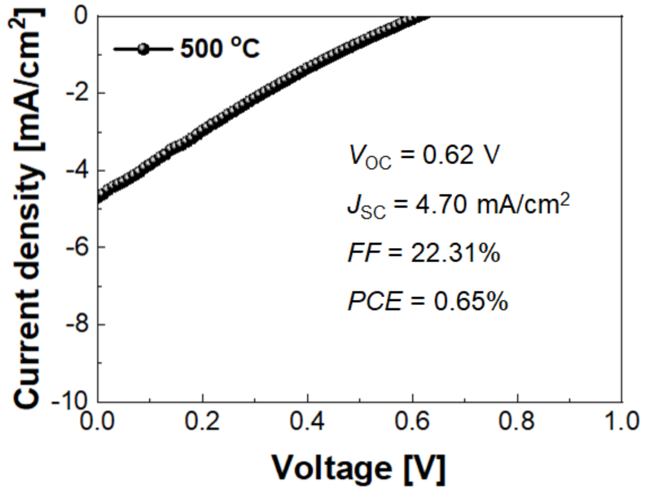

Figure S5. *J*–*V* curve of the device employing $ZrSnO_4$ (500 °C) ETL.

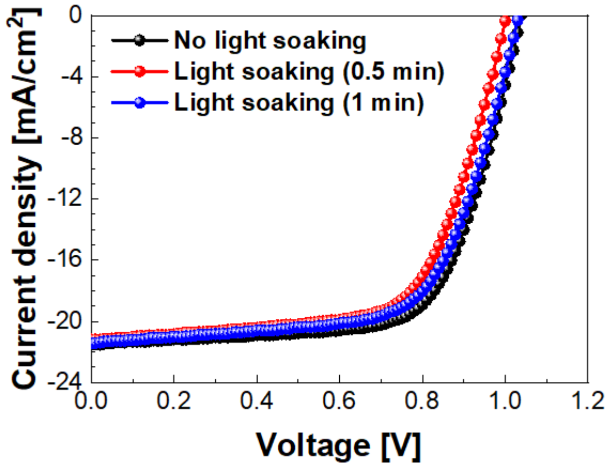

Figure S6. *J*–*V* curves of the devices employing $ZrSnO_4$ (400 °C) ETL under different light soaking.
